# Polyphenol Bioavailability and Plasma Antiradical Capacity in Healthy Subjects after Acute Intake of Pigmented Rice: A Crossover Randomized Controlled Clinical Trial

**DOI:** 10.3390/jcm9103209

**Published:** 2020-10-05

**Authors:** Sara Vitalini, Andrea Sardella, Daniela Fracassetti, Rita Secli, Antonio Tirelli, Giovanni Lodi, Antonio Carrassi, Elena Maria Varoni, Marcello Iriti

**Affiliations:** 1 Department of Agricultural and Environmental Sciences, Università degli Studi di Milano, 20133 Milan, Italy; sara.vitalini@unimi.it; 2 Department of Biomedical, Surgical and Dental Sciences, Università degli Studi di Milano, 20142 Milan, Italy; andrea.sardella@unimi.it (A.S.); giovanni.lodi@unimi.it (G.L.); antonio.carrassi@unimi.it (A.C.); 3 ASST Santi Paolo e Carlo–Presidio Ospedaliero San Paolo–Università degli Studi di Milano, 20142 Milan, Italy; rita.secli@asst-santipaolocarlo.it; 4 Department of Food, Environmental and Nutritional Sciences, Università degli Studi di Milano, 20133 Milan, Italy; daniela.fracassetti@unimi.it (D.F.); antonio.tirelli@unimi.it (A.T.)

**Keywords:** cereals, black rice cultivars, flavonoids, anthocyanins, antioxidant activity

## Abstract

Health-promoting effects of plant foods have been emphasized in the last few decades and ascribed to the bioactive phytochemicals present therein—in particular, phenylpropanoids. The latter have been investigated for a number of preclinical biological activities, including their antioxidant power. Due to the paucity of human studies, in this randomized intervention trial, we investigated whether the acute intake of pigmented rice could increase the plasma bioactive levels and antiradical power in twenty healthy subjects. A crossover randomized controlled clinical trial was conducted on 19 volunteers. Artemide and Venere black rice cultivars were tested, while brown rice Carnaroli was used as a control. Each patient received randomly one serving (100 g) of rice on three different experimental days, separated by a 7-day washout period. After baseline blood withdrawal, time-course changes of plasma polyphenols, flavonoids and radical-scavenging capacity were determined at 30, 60, 120 and 180 min post rice intake. Compared to Carnaroli rice, the two black rice cultivars significantly increased the plasma levels of polyphenols and flavonoids at 60 and 120 min and, correspondingly, the plasma antiradical power at 60 min after consumption. Pigmented rice consumption can contribute to diet-related health benefits in humans.

## 1. Introduction

Polyphenols are a large group of bioactive phytochemicals arising from the aromatic amino acid phenylalanine and consisting of flavonoids, stilbenes and proanthocyanidins [1]. In the last few decades, foods rich in these plant secondary metabolites, including pigmented cereals, have been extensively investigated for their preclinical biological and pharmacological activities, with many studies focusing on the antioxidant capacity [2,3]. In addition, as recently reported, pigmented cereal extracts exhibited anti-inflammatory activity and inhibited pancreatic α-amylase and intestinal α-glucosidase in vitro in a dose-dependent manner [4].

Both observational and interventional studies have also emphasized the health-promoting effects of dietary styles rich in plant-based foods, in terms of longevity, healthy ageing and morbidity [2,3]. Acute consumption of pigmented rice varieties increased plasma antioxidant and anti-inflammatory activities in 24 healthy subjects [5]. In a meta-analysis of 128 randomized clinical trials involving 5438 participants, polyphenol-rich food consumption reduced total cholesterol and blood pressure, while increasing vasodilatation, particularly in obese/overweight individuals [6]. Another recent meta-analysis of 32 randomized clinical trials showed that dietary polyphenols reduced systemic and vascular inflammation by modifying the levels of specific biomarkers [7].

However, the content of polyphenols in plant foods (not consumed raw), such as rice, still needs to be better elucidated and strictly depends on cooking method; moreover, their health benefits depend on oral bioavailability. In a previous study, we showed that, among the cooking methods investigated, the rice cooker mostly preserved the polyphenol content and antiradical capacity of pigmented rice cultivars [8]. Therefore, this randomized clinical trial aimed at investigating whether acute pigmented rice consumption could increase the plasma polyphenol levels and antiradical capacity in healthy volunteers.

## 2. Experimental Section

### 2.1. Chemicals and Reagents

Standards of gallic acid, sodium acetate, 2,2′-azino-bis(3-ethylbenzothiazoline-6-sulphonic acid) (ABTS), 2,2-diphenyl-1-picrylhydrazyl (DPPH), Folin–Ciocalteu reagent, 6-hydroxy-2,5,7,8-tetramethylchroman-2-carboxylic acid (Trolox) and hydrochloric acid were purchased from Sigma-Aldrich (Darmstadt, Germany). Catechin was purchased from Extrasynthese (Genay, France). Ethanol and methanol were purchased from Novachimica (Milano, Italy).

### 2.2. Rice Samples and Cooking

Rice samples were provided by SA.PI.SE. Coop. Agr. (Vercelli, Italy) and consisted of two black (Venere and Artemide) and one brown (Carnaroli) varieties. They were chosen based on a previous study [8] and because they are widely consumed in Northwest Italy. Before the dietary intervention trial, all rice samples were stored at 4 °C. They were cooked in a standard domestic rice cooker at water:rice ratio 2:1 for 25 min, as previously reported [8].

### 2.3. Recruitment of Volunteers, Eligibility Criteria and Setting

From December 2018 to April 2019, participants were recruited from students of the University of Milan (Italy), including female and male young Caucasian individuals (20–35 years), who were healthy, with normal weight (Body Mass Index, BMI, 18.5–25.0 kg m^−2^), who voluntarily agreed to join the study. Exclusion criteria were pregnancy and lactation; taking dietary supplements (vitamins, antioxidants, botanicals, phytochemicals) and drugs; abnormal hematological parameters; heavy smoking and heavy alcohol drinking; high-intensity physical activity. For each subject, demographics, anthropometric characteristics and dietary habits were recorded. After initial participant screening, individuals considered eligible were contacted. All volunteers provided their written and signed informed consent. All data, including blood samples, were collected at the Dental Clinic-Odontostomatologia II Unit (ASST Santi Paolo e Carlo–Presidio Ospedaliero San Paolo–Milan State University, Italy).

### 2.4. Trial Design Including Allocation

The study was designed as a crossover, randomized, controlled dietary intervention trial. CONSORT guidelines were followed to report the trial. The study was conducted in accordance with the ethical principles of the Declaration of Helsinki, received the approval by the institutional Ethical Committee (MI 17/07/2018) and was registered at www.clinicaltrial.gov (ID: NCT03935022). A simple randomization method was applied by an allocation software program (http://graphpad.com/quickcalcs/randomise1.cfm), which generated five different random sequences of treatment assignment (Table 1); each of them was composed of 4 individuals. Allocation concealment was assured because the person who generated the randomization and assigned the volunteers to the three arms was not involved in evaluating the eligibility of individuals and their enrolment or in any further phase of the clinical trial. The treatments were black rice Venere, black rice Artemide and brown rice Carnaroli (control). Participants were thus enrolled, randomized and allocated to the specific sequence of interventions by two further investigators. Each patient received one serving (100 g) of rice on three different experimental days separated by a 7-day washout period, according to the sequences reported in Table 1.

The content of bioactive phytochemicals in a rice portion and its antiradical capacity were estimated based on Fracassetti et al. [8] (Table 2).

Volunteers were deprived of polyphenol-rich food sources three days before experimentation and during each washout period. Participants received a complete list of foods to be avoided. Breakfast, lunch and dinner were standardized one day before the experiment. For all interventions, after the overnight fasting, in a quiet room of the Dental Clinic, participants consumed the 100-g rice serving (including the cooking water) within 10–15 min, immediately after the first basal blood collection (10 mL) at 8:00 a.m. (baseline), under the direct supervision of investigators. The subsequent blood withdrawals were collected at 30, 60, 120 and 180 min post rice consumption. The volunteers were instructed to maintain low physical activity for the duration of the study and to maintain hydration by consuming 250 mL of water every 30 min.

### 2.5. Blood Sample Preparation

Withdrawals of blood were collected by venipuncture and immediately centrifuged (2000 rpm for 20 min at 20 °C; Hettich, Tuttlingen, Germany) to separate the plasma from the blood cells. The centrifuged samples were stored at −80 °C until extraction and analysis. The samples were thawed at room temperature and the extraction was carried out following the procedure reported by Serafini et al. [9] with some modifications. Then, 100 μL of thawed plasma was added with 200 μL of 1 M hydrochloric acid, vortexed for 1 min and incubated in a water bath at 37 °C for 30 min. Two hundred μL of 2 M sodium hydroxide in 75% methanol (v/v) was then added, vortexed for 1 min and incubated in a water bath at 37 °C for 30 min. The last step was the addition of 0.74 M orthophosphoric acid (200 μL); the tubes were vortexed for 3 min and centrifuged at 8000× *g* for 10 min at 5 °C (Hettich, Tuttlingen, Germany). The supernatant was recovered and stored at 4 °C. The pellet was resuspended in 200 μL of acetone/water 50/50 (v/v) and vortexed for around 3–5 min. The sample was centrifuged at 8000× *g* for 10 min at 5 °C; the supernatant was jointly collected. The extraction was carried out in triplicate.

### 2.6. Outcomes—Polyphenol Bioavailability and Antiradical Capacity

Investigators who performed polyphenol bioavailability and antiradical capacity analyses were blind to interventions.

#### 2.6.1. Determination of Total Phenol Index

The total phenol index (TPI) was spectrophotometrically determined by absorbance reading at 280 nm. The samples were properly diluted with water, allowing the reading of an absorbance value lower than 1 AU. The readings were carried out in triplicate for each extract. TPI was determined by multiplying the absorbance value at 280 nm for the sample dilution.

#### 2.6.2. Determination of Total Polyphenols

Total polyphenols were spectrophotometrically determined by means of the Folin–Ciocalteu reagent (a colorimetric assay) [10]. The reagent was diluted 10 times in water (v/v) (2.5 mL) and added to 0.5 mL of sample. Two milliliters of 75 g L^−1^ sodium carbonate solution was added and the tubes were kept for 1 h at room temperature in the dark. The absorbance at 765 nm was measured and the results were expressed as µg gallic acid/mL of plasma. The calibration curve for gallic acid (5–100 mg L^−1^) was dissolved in methanol/water 50/50 (v/v) [11].

#### 2.6.3. Determination of Total Flavonoids

Total flavonoids were determined after sample dilution in hydrochloric ethanol (ethanol/water/hydrochloric acid 37% 70/30/1 v/v/v). The quantification was carried out following the procedure reported by Di Stefano et al. [12]. The readings were carried out in triplicate for each extract. Data were expressed in μg catechin/mL of plasma.

#### 2.6.4. Determination of Antiradical Capacity

The antiradical power was determined by DPPH and ABTS assays after properly diluting the extracts in methanol/water 70/30 (v/v). The DPPH radical scavenging assay was carried out following the method of Brand-Williams et al. [13] with some modifications as reported by Fracassetti et al. [11]. The ABTS radical scavenging capacity was determined according to Vitalini et al. [14]. Reaction mixtures were carried out in triplicate and data were expressed as μM Trolox mL^−1^ of plasma.

### 2.7. Statistical Analysis

The sample size (N = 18) to ensure at least 80% statistical power (α level set at 0.05) was calculated, using online statistical software (http://hedwig.mgh.harvard.edu/sample_size/js/js_crossover_quant.html). The final number was set to 22 considering a potential 10% patient dropout from the study. Statistical analysis was performed on blinded data. Factorial ANOVA was carried out using SPSS statistical software (IBM SPSS Statistics 24, International Business Machines Corporation; Armonk, NY, USA) considering the rice variety and the time after consumption as continuous variables. The homogeneity of the variance was evaluated with Levene test and significant differences among samples were determined with Fischer’s test (least significant difference, LSD). Differences were significant for *p* < 0.05 and *p* < 0.01. Results were expressed as percentage of increase from baseline (T = 0), set as 100%.

## 3. Results

**Figure 1:**
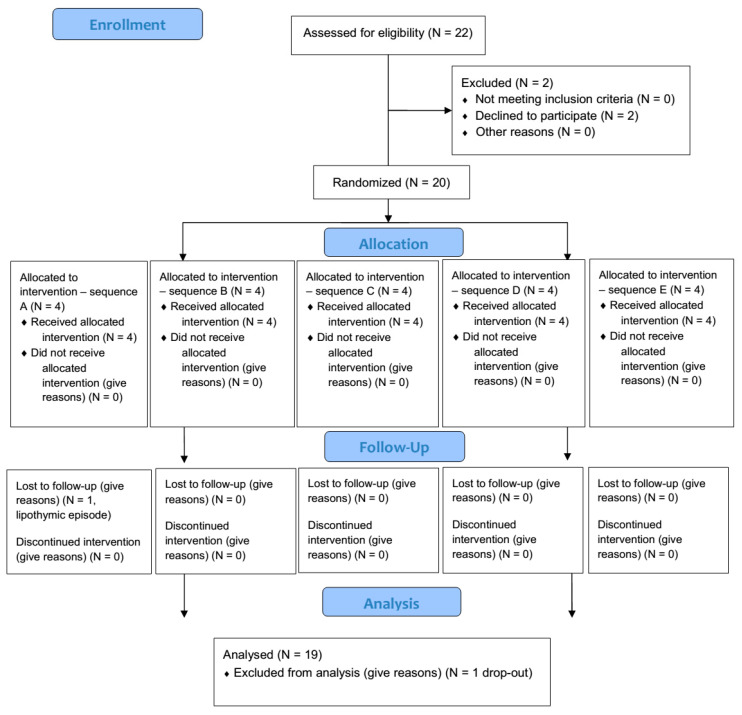
CONSORT (consolidated standards of reporting trials) flowchart of the study design.

Twenty-two healthy subjects were assessed for eligibility (12 males and 10 females). Since two of them refused to participate, twenty volunteers were finally enrolled (11 males and 9 females; mean age: 24.7 ± 3.8 years; mean BMI: 21.9 ± 2.1 kg m^−2^). Nineteen out of 20 participants completed the study with high compliance; only one female volunteer dropped out because of a lipothymic episode during the blood withdrawal at baseline (CONSORT flowchart, Figure 1).

**Figure 2:**
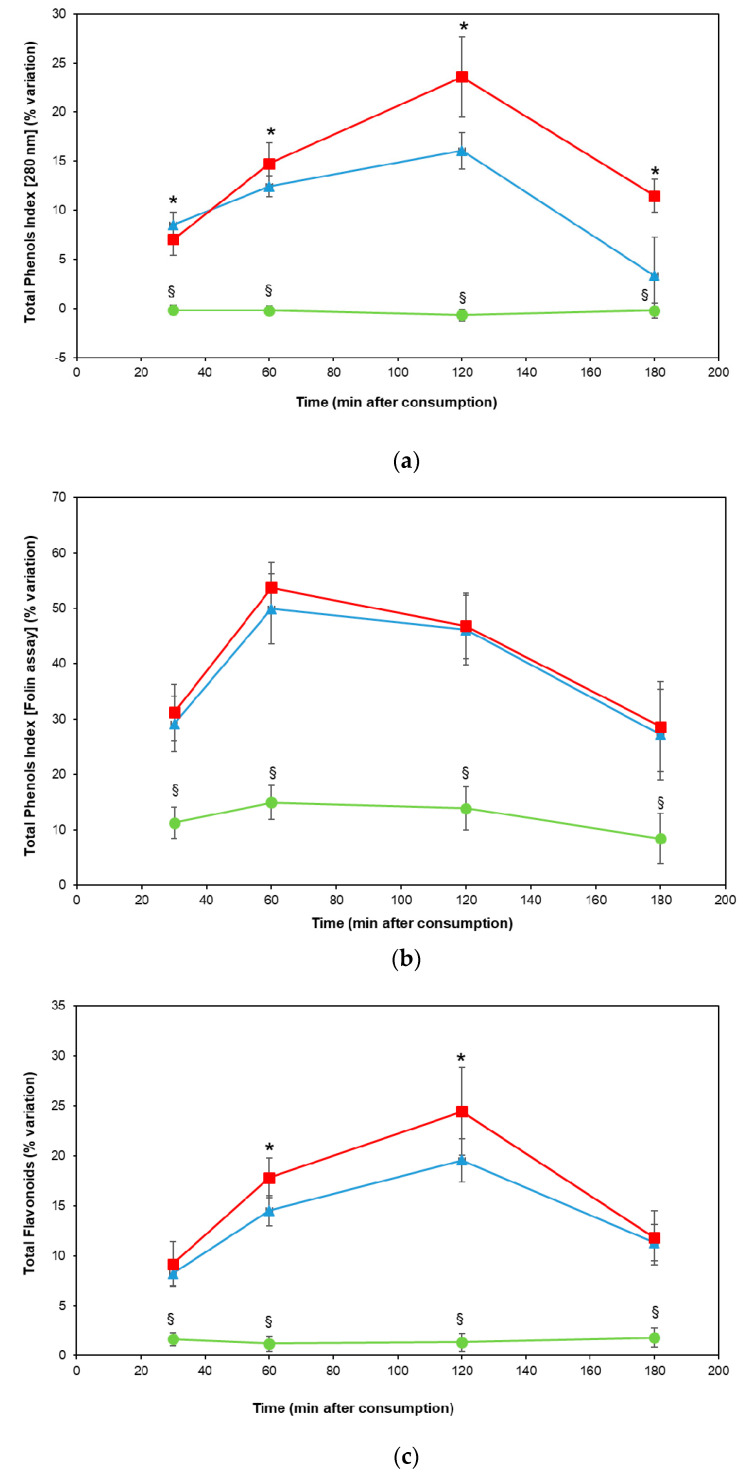
(**a**) Percentage variation (mean ± standard error) in the plasma total phenol index (spectrophotometric assay, absorbance reading at 280 nm) of volunteers (N = 19) 30, 60, 120 and 180 min after consuming 100 g of Venere (square), Artemide (triangle) or Carnaroli (circle) rice. The symbol (§) indicates statistically significant differences between Carnaroli rice and the pigmented varieties (*p* < 0.01); the symbol (*) indicates statistically significant differences between Venere and Artemide rice (*p* < 0.05); (**b**) percentage variation (mean ± standard error) in the total polyphenol plasma levels (Folin–Ciocalteu colorimetric assay, absorbance reading at 765) of volunteers (N = 19) 30, 60, 120 and 180 min after consuming 100 g of Venere (square), Artemide (triangle) or Carnaroli (circle) rice. The symbol (§) indicates statistically significant differences between Carnaroli rice and the pigmented varieties (*p* < 0.01); **(c)** percentage variation (mean ± standard error) in the total flavonoid plasma levels (spectrophotometric assay) of volunteers (N = 19) 30, 60, 120 and 180 min after consuming 100 g of Venere (square), Artemide (triangle) or Carnaroli (circle) rice. The symbol (§) indicates statistically significant differences between Carnaroli rice and the pigmented varieties (*p* < 0.01); the symbol (*) indicates statistically significant differences between Venere and Artemide rice (*p* < 0.05).

After pigmented rice consumption, a significant increase in plasma total phenol index was observed from baseline up to 120 min, for both cultivars, compared with brown rice (Carnaroli) (*p* < 0.01) (Figure 2A). The cultivar Venere exhibited a higher increase in plasma total phenol index with respect to Artemide, the other pigmented cultivar (*p* < 0.05) (Figure 2A). A peak in plasma total polyphenols was measured with the colorimetric Folin–Ciocalteu assay 60 min post pigmented rice intake (*p* < 0.01) (Figure 2B). Similar to the total phenol index, administration of pigmented rice increased the plasma total flavonoids, with a maximum at 120 min, as compared to Carnaroli rice (*p* < 0.01) (Figure 2C). The cultivar Venere showed a higher increase in plasma total flavonoids than the Artemide one (*p* < 0.05) (Figure 2C).

**Figure 3:**
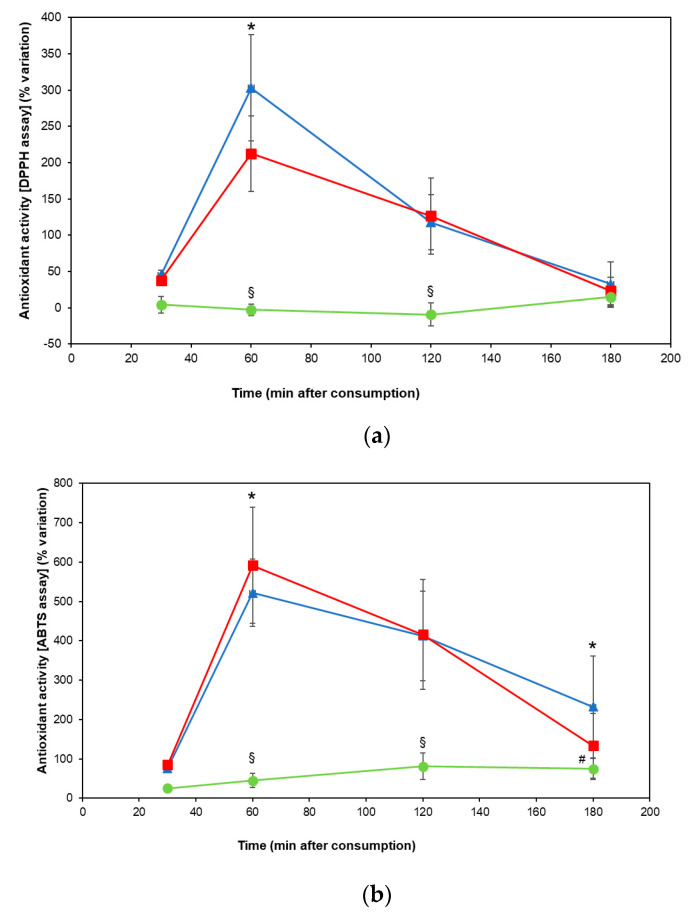
(**a**) Percentage variation (mean ± standard error) in the plasma antiradical capacity (DPPH radical scavenging assay) of volunteers (N = 19) 30, 60, 120 and 180 min after consuming 100 g of Venere (square), Artemide (triangle) or Carnaroli (circle) rice. The symbol (§) indicates statistically significant differences between Carnaroli rice and the pigmented varieties (*p* < 0.01); the symbol (*) indicates statistically significant differences between Venere and Artemide rice (*p* < 0.05). (**b**) Percentage variation (mean ± standard error) of the plasma antiradical capacity (ABTS radical scavenging assay) of volunteers (N = 19) 30, 60, 120 and 180 min after consuming 100 g of Venere (square), Artemide (triangle) or Carnaroli (circle) rice. The symbol (§) indicates statistically significant differences between Carnaroli rice and the pigmented varieties (*p* < 0.01); the symbol (#) indicates statistically significant differences between Carnaroli and Artemide rice (*p* < 0.05); the symbol (*) indicates statistically significant differences between Venere and Artemide rice (*p* < 0.05).

Both DPPH and ABTS assays indicated that pigmented rice consumption significantly increased the plasma antiradical capacity in volunteers, in comparison with brown rice, showing a peak at 60 min (*p* < 0.01) (Figure 3A,B). The intake of Artemide rice caused higher plasma DPPH radical scavenging power than the cultivar Venere (*p* < 0.05) (Figure 3A), whereas plasma samples measured by ABTS assay showed that Venere rice increased antiradical capacity more than the cultivar Artemide (*p* < 0.05) (Figure 3B).

## 4. Discussion

Despite the common opinion agreeing that pigmented cereals are healthy, randomized clinical trials investigating the effects of black rice consumption in humans are still scanty. The results of our dietary intervention study have demonstrated that acute intake of black rice cultivars significantly increase the plasma antiradical capacity in healthy volunteers, probably due to the bioavailable polyphenols (including flavonoids) from consumed pigmented rice. Indeed, as previously reported, Venere and Artemide cultivars are rich in polyphenols [8] that may synergistically contribute to a rise in plasma antioxidant activity, with possible anti-inflammatory effects [5].

A peak in total flavonoid absorption, in particular, was recorded 120 min after pigmented rice administration. Likewise, the total phenol index showed a similar kinetics of absorption. However, the determination of total polyphenols showed an earlier plasma peak, i.e., 60 min after ingestion. This difference could be attributed to the intrinsic diversity between the two spectrophotometric assays. Indeed, the Folin–Ciocalteu colorimetric method, expressed as equivalents of gallic acid (absorbance at 765 nm), could measure phenylpropanoids absorbed more quickly, compared to the total phenol index (absorbance at 280 nm).

With regard to differences between the pigmented rice cultivars, the plasma levels of the bioactive compounds were always higher in the volunteers after Venere cultivar consumption, although the latter had a lower content of total polyphenols and flavonoids than Artemide rice. Probably, the phenolic compounds of Venere rice could be better absorbed by subjects than those present in Artemide cultivar, albeit to a greater extent. Nonetheless, it should be kept in mind that the applied methods (total phenol index, total polyphenols and total flavonoids) are only indexes providing general information about polyphenol absorption and bioavailability in humans.

In our experimental conditions, both DPPH and ABTS radical scavenging assays showed peak activity 60 min after the administration of pigmented rice. In the most unique study available to date investigating the acute antioxidant effects of pigmented rice in a healthy population, plasma antioxidant activity (measured by the ferric reducing ability of plasma) increased 30 min after purple rice consumption and remained elevated for all time points (60, 120 and 240 min) [5].

The highest increase in the antiradical capacity was measured after the ingestion of Artemide rice, in the case of the DPPH assay, while, for the ABTS assay, the consumption of Venere rice determined the highest values of activity. This difference could be due to the diverse phytochemical composition of the pigmented rice cultivars [15]. Therefore, the different content of water- and lipid-soluble compounds in the cultivars could determine their diverse reactivity towards the chromophores used for the test, the radical DPPH and the cation radical ABTS [16,17,18]. We can speculate that the maximum increase in antioxidant capacity is due to these compounds being absorbed faster by volunteers, i.e., those determined with the Folin–Ciocalteu colorimetric assay and with a maximum peak at 60 min.

However, other compounds such as vitamin E and γ-oryzanol, which have not been measured in this study, may be present in rice and contribute to its antioxidant potential. Therefore, plasma antioxidant activity cannot be uniquely attributed to the phenolic content of rice but also to the actions of different antioxidant compounds present in the latter as well as endogenous antioxidant systems present in plasma (such as uric acid or glutathione), possibly stimulated upon intake and with possible synergistic or additive effects which are still unknown. Notably, DPPH and ABTS assays detect not only polyphenols but all scavengers of these synthetic radicals.

Finally, although the conclusions to be drawn from these results in relation to the direct health effects of pigmented rice in healthy individuals need to be further investigated, the increase in plasma antioxidant activity is here demonstrated and could have some pathophysiological significance, oxidative stress being involved in the etiopathogenesis of important chronic-degenerative diseases [2,3]. The limitations of this trial include the short-term intake of rice, not enough to evaluate long-term health effects, and the enrollment of university students as volunteers (selection bias). The young age of participants and the setting where the intervention was provided (in a hospital, at breakfast) may hinder the generalizability of the trial findings. Therefore, it would be relevant to evaluate the effects of chronic intake of pigmented rice for no less than three weeks on a larger sample of a healthy population, including older patients, in a setting closer to real life. In such a direction, a recent meta-analysis of randomized clinical trials indicates that habitual consumption of pigmented foods can improve the low-grade chronic inflammatory state typically found in cardiovascular disease [7].

## 5. Conclusions

In this study, we showed that the acute intake of black rice significantly increased the plasma levels of polyphenols and flavonoids and, correspondingly, the plasma antiradical power in healthy subjects. Time-course changes in plasma polyphenols, flavonoids and radical-scavenging capacity were determined at 30, 60, 120 and 180 min post rice intake. The two black rice cultivars significantly increased the plasma levels of polyphenols and flavonoids at 60 and 120 min and, correspondingly, the plasma antiradical power at 60 min after consumption. Pigmented rice consumption can contribute to diet-related health benefits in humans. Further studies are needed to elucidate the benefits of long-term intake of black rice cultivars, involving a larger population.

## Figures and Tables

**Table 1:** Sequential steps of study interventions.

Sequence	Washout (days)	Rice Administered (1st Experimental Day)	Washout (days)	Rice Administered (2nd Experimental Day)	Washout (days)	Rice Administered (3rd Experimental Day)
A	7	Venere	7	Artemide	7	Carnaroli
B	7	Carnaroli	7	Artemide	7	Venere
C	7	Venere	7	Carnaroli	7	Artemide
D	7	Artemide	7	Venere	7	Carnaroli
E	7	Carnaroli	7	Venere	7	Artemide

**Table 2:** Total polyphenols, total flavonoids, total anthocyanins and antiradical capacity of a serving (100 g) of rice (Artemide, Venere and Carnaroli) after cooking in rice cooker for 25 min, including the cooking water (estimated according to Fracassetti et al., 2020 [8]).

Rice	Total Polyphenols (mg gallic acid/100 g)	Total Flavonoids (mg catechin/100 g)	Total Anthocyanins (mg cyanidin/100 g)	Antiradical Capacity (µM Trolox/100 g)	
				DPPH ^ Assay	ABTS * Assay
Venere	149 ± 13 ^§^	240 ± 13	711 ± 76	701 ± 90	2146 ± 68
Artemide	806 ± 54	545 ± 31	614 ± 34	4366 ± 736	5397 ± 432
Carnaroli	29 ± 3	nd	nd	nd	20 ± 2

^§^ Results are expressed as mean ± standard deviation; ^ 2,2-diphenyl-1-picrylhydrazyl; * 2,2′-azino-bis(3-ethylbenzothiazoline-6-sulphonic acid).
